# Segmental Tissue Speckle Tracking Predicts the Stenosis Severity in Patients With Coronary Artery Disease

**DOI:** 10.3389/fcvm.2021.832096

**Published:** 2022-02-03

**Authors:** Srisakul Chaichuum, Shuo-Ju Chiang, Masao Daimon, Su-Chen Chang, Chih-Lin Chan, Chu-Ying Hsu, Hsiang-Ho Chen, Ching-Li Tseng

**Affiliations:** ^1^Graduate Institute of Biomedical Materials and Tissue Engineering, Taipei Medical University, Taipei, Taiwan; ^2^Division of Cardiology, Department of Internal Medicine, Taipei City Hospital Yangming Branch, Taipei, Taiwan; ^3^Department of Cardiovascular Medicine, The University of Tokyo Hospital, Tokyo, Japan; ^4^Center for Biomedical Engineering, College of Engineering, Graduate Institute of Biomedical Engineering, Chang Gung University, Taoyuan, Taiwan

**Keywords:** coronary angiography, stenosis rate, tissue speckle tracking, echocardiography, coronary artery disease

## Abstract

**Objective:**

Two-dimensional speckle tracking echocardiography (2D-STE) has been used as a diagnostic tool for coronary artery disease (CAD). However, whether vessel supplied myocardial strain and strain rate (SR) predict the severity of coronary artery stenosis in patients with CAD is unknown. This study aimed to investigate correlation of cardiac mechanical parameters in tissue speckle tracking measurements with coronary artery stenosis diagnosed by cardiac catheterization in patients with clinically diagnosed CAD.

**Methods and Results:**

Among 59 patients analyzed, 170 vessels were evaluated by coronary angiography and the corresponding echocardiography to quantify left ventricular myocardial strain and SR. The average longitudinal strain and SR of the segmental myocardium supplied by each coronary artery were calculated to achieve vessel myocardium strain (VMS) and strain rate (VMSR). The VMS and VMSR at each of four severity levels of stenosis showed significant differences among groups (*p* = 0.016, and *p* < 0.001, respectively). The strain and SR in vessels with very severe stenosis (≥75%, group IV; *n* = 29), 13.9 ± 4.3, and 0.9 ± 0.3, respectively, were significantly smaller than those of vessels with mild stenosis ≤ 25%, group I; *n* = 88, 16.9 ± 4.9, *p* = 0.023, and 1.2 ± 0.3, *p* = 0.001, respectively. The SR in vessels with moderate stenosis (26–49%, group II; *n* = 37), 1.0 ± 0.2, was significantly smaller than that in vessels with mild stenosis vessels (*p* = 0.021). The lower VMS and VMSR, the higher possibility of severe coronary stenosis is. The VMS and VMSR lower than 13.9 ± 4.3 and 0.9 ± 0.3, respectively predicted the severe coronary stenosis. The VMS and VMSR higher than 16.9 ± 4.9 and 1.2 ± 0.3, respectively predicted mild or no coronary artery stenosis.

**Conclusions:**

The actual stenosis rate in catheterization demonstrates that this technique was able to assess coronary artery condition. Thus, the application of a non-invasive method of 2D-STE to evaluate and simplify diagnosis of CAD is feasible.

## Introduction

It is widely known that cardiovascular disease (CVD) is a leading cause of mortality, and CVD has affected the economy worldwide. According to a report from the American Heart Association in Heart Disease and Stroke 2021 Statistics Update, ~18.6 million people died from CVD in 2019. In the US, the estimated direct and indirect economic cost of heart disease from 2016 to 2017 was $219.6 billion (1). Coronary artery disease (CAD), a major adverse cardiac event, is the underlying cause of angina, myocardial infarction, and sudden cardiac death. Despite various advances in technology, early diagnosis of CAD remains challenging. Moreover, tissue level cardiac mechanics are not understood systematically. Although coronary angiography is the gold standard method for identification of coronary artery stenosis, catheterization is invasive compared to other diagnostic tools (2). Echocardiography is the most accessible and cost-effective technique routinely used for these patients. This non-invasive imaging model has been utilized visually across the spectrum of CAD (3). Emerging ultrasound procedures, such as tissue Doppler imaging and the speckle tracking method, are gradually becoming required for first-line clinical evaluation. However, the disease process of myocardial mechanics occurs much earlier before the structural changes of the myocardium are expressed and visualized *via* traditional echocardiography (4). In terms of technical aspects, with improvement of echocardiographic processing, traditional Doppler imaging has evolved to the tissue level. To measure myocardial tissue velocity in the Doppler ultrasound system, a high-pass filter is implemented to exclude the low-frequency components from the vessel wall and the blood flow signal. However, this technique could lead to a potential loss of information from the low-velocity flow (5, 6). Speckle tracking echocardiography (STE) is a novel extension of the Doppler technique that focuses on left ventricular (LV) wall motion. LV wall motions represent the major cardiac events that occur in all individuals. Three main coronary arteries have their major distribution in this area. Two-dimensional speckle tracking echocardiography (2D-STE) has been determined to be a promising tool for LV functional assessment and can detect subclinical myocardial dysfunction early in the disease process (7, 8). Computer algorithms evaluate the fractional or percent change of “speckle” observed from the original dimension of the myocardium to compute the strain, deformation, dimensionless quantity, and strain rate (SR), which is the change in strain over time (9). Strain and SR are superior to the velocity measurement of tissue Doppler, which is inherently angle dependent (10). In 2D-STE, SR is a good representation of myocardial contractility and rate of change in pressure (dP/dt). Experimental studies have demonstrated that the degree of post-systolic thickening and SR are suitable parameters for ischemia detection (11, 12).

Here, we hypothesized that the regional strain and SR of vessels, specifically in segmental lesions, can be used to predict the stenosis condition in each coronary artery of patients with CAD. Thus, this study aimed to investigate potential correlations among strain, SR and coronary artery stenosis diagnosed by cardiac catheterization.

## Methods

### Patient Selection

The cross-sectional study was conducted. We recruited 59 patients with suspected CAD in Taipei City Hospital Yangming Branch as selection criteria by clinical diagnosis from August 2020 to May 2021. Patients with clinically diagnosed CAD assessed by transthoracic echocardiography (TTE) were included. Individuals aged <20 years and >75 years were excluded. Patients with terminal major organ disease, cancer, hemodialysis, significant valvular disease or a prosthetic valve, any rhythm other than sinus (including atrial and ventricular arrhythmias and pacemaker rhythm), an unstable hemodynamic condition, obesity with BMI ≥30 kg/m^2^ (13–15), and inadequate quality ultrasound images were excluded. Patients with a left ventricular ejection fraction (LVEF) <55% and regional wall motion abnormality (RWMA) were also excluded.

### Echocardiographic Examination

All patients underwent comprehensive TTE by experienced sonographers, within 2–3 weeks before coronary angiography. The machine used for evaluation was a commercially available system (Vivid E95, GE Healthcare, Horten, Norway) equipped with M5S, a 1.4–4.6 MHz phased array probe. In all subjects, standard 2D images, the apical 4-, 3-, and 2-chamber views (A4C, A3C, and A2C, respectively), consisting of three cardiac cycles actuated to the QRS complex, were captured and analyzed. Conventional 2-D parameters were analyzed and recorded (16). LVEF was calculated by Simpson's biplane method. Preserved and normal LVEF were indicated as 50–60% and >60%, respectively. LV diastolic function was also assessed according to the guideline (17). Peak velocities of early (E) and late (A) diastolic flow, and the E/A ratio were measured using pulsed-wave interrogation of the mitral valve inflow. Tissue Doppler imaging was performed to assess septal mitral annular motion. Early septal diastolic annular velocity (e') based on tissue Doppler imaging was measured. The E/e' ratio was calculated (18).

### 2D Speckle Tracking

**Figure 1:**
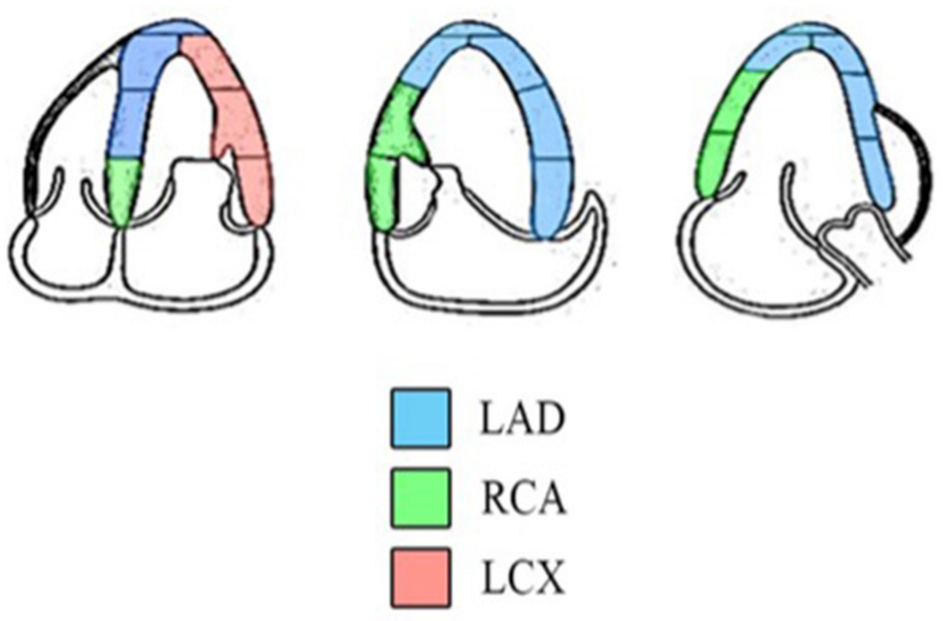
Diagram indicating the distributions of coronary artery perfusion. The myocardium is supplied by the LAD, RCA, and LCX (18).

**Figure 2:**
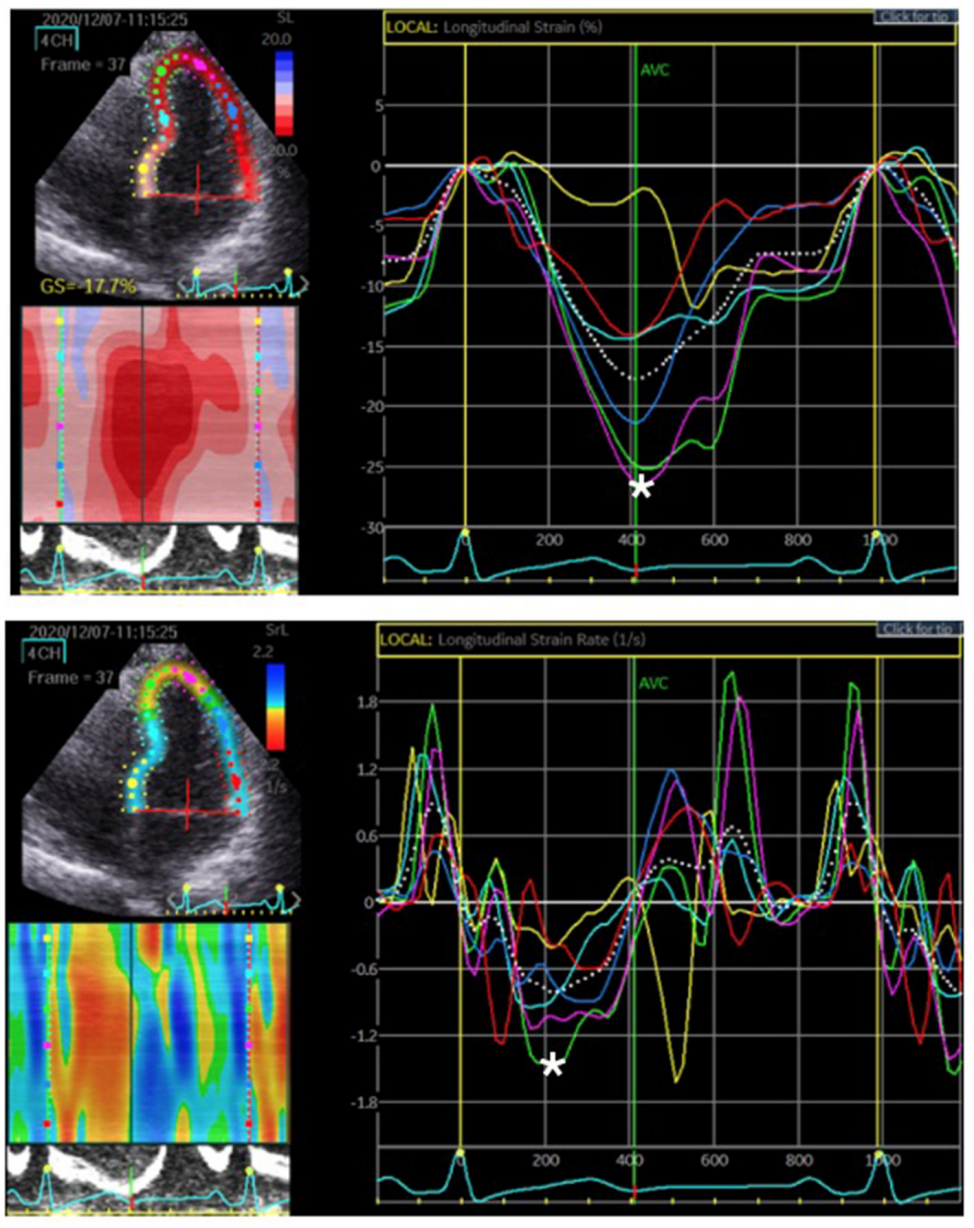
The region of interest, LV myocardium, was tracked. The longitudinal strain and SR were quantified as the peak systolic value in each myocardial segment. The upper figure represents the peak systolic strain. The most negative value, which is denoted by the white asterisk, was measured before aortic valve closure (AVC). The lower figure represents the peak systolic SR. The most negative value is denoted by the white asterisk on the rate/time curve during systole.

The standard echocardiographic views in A4C, A3C, and A2C on three consecutive beats were used to obtain longitudinal strain and SR analysis of the LV using 2D speckle tracking software (EchoPAC, GE Healthcare, Horten, Norway). Adequate TTE examinations, defined as good image quality and an optimum frame rate of 50–70 *frames per second, were used for* feature myocardial tracking. The myocardium was divided into six segments in each view. The recorded images were analyzed offline. A region of interest was defined at end-diastole by manual outline. Individual regions of the border were adjusted until the whole myocardium was correctly tracked. The wall thickness was adjusted manually if necessary for complete analysis (19). Myocardial strain is a measure of the deformation when two neighboring points of myocardium move at different velocities resulting in myocardium changing its shape (deforming). Strain can be presented as percentage (%), which includes lengthening (positive strains) and shortening (negative strains). Myocardial SR is the rate of deformation in the unit of 1/s. Cardiac strain and SR were assessed to evaluate myocardial deformation and displayed in an 18-segment LV model. Three main arteries, the right coronary artery (RCA), the left circumflex artery (LCX), and the left anterior descending artery (LAD), supply the myocardium. The regional wall segments in a series of longitudinal views were composed of basal septal, mid septal, apical septal, apical lateral, mid lateral, and basal lateral segments of the LV. According to the American Heart Association recommendations for use of echocardiography (20), the distributions of coronary artery perfusion are described in Figure 1. The basal septal LV segment in A4C, basal and mid septal LV segments in A3C, and basal and mid septal LV segments in A2C are supplied by the RCA. The apical, mid, and basal lateral LV segments in A4C are supplied by the LCX. The mid and apical septal LV segments in A4C, apical septal and apical, mid, and basal lateral LV segments in A3C, and apical septal and apical, mid, and basal lateral LV segments in A2C are supplied by the LAD (20). The myocardium of the 2D-STE images of each of the three coronary arteries was quantified to obtain the longitudinal strain and SR of each wall, as shown in Figure 2. During systole and diastole, the myocardium shortens and lengthens the wall muscles in the longitudinal planes. The average longitudinal strain and SR of the segmental myocardium supplied by each coronary artery were calculated to achieve vessel myocardium strain (VMS) and strain rate (VMSR). Inter-observer reproducibility was also examined.

Coronary artery disease was diagnosed by clinical symptoms, electrocardiography, treadmill exercise stress test, thallium scan and cardiac CT. Common medications used in patients with CAD, such as nitrates, β-blockers and statins, were reported.

### Cardiac Catheterization

The study population was categorized by the severity of coronary artery stenosis diagnosed by coronary angiography. Stenosis was considered significant if there was ≥70% diameter stenosis according to the indication for percutaneous coronary intervention (PCI) (21). The luminal stenosis of the three main arteries, RCA, LCX and LAD, was quantified for further evaluation of CAD. The stenosis condition was categorized into four severity levels, ranging from mild to very severe. Cases with ≤ 25% stenosis were classified as mild (group I); cases with 26–49% stenosis were classified as moderate (group II); cases with 50–74% stenosis were classified as severe (group III); cases with ≥75% stenosis were classified as very severe (group IV) following criteria modified from Arbab-Zadeh and Fuster (22, 23).

Among the patients analyzed, 170 vessels were evaluated by coronary angiography and 2D-STE. The results of vessel-supplied tissue tracking and coronary angiography were compared.

### Statistical Analysis

All continuous variables are presented as mean ± standard deviation, whereas categorical variables are presented as proportions or percentages. Comparison of continuous variables within two groups was performed using an independent *t*-test. Comparison of continuous variables between coronary artery stenosis severity groups was performed using one-way ANOVA with Tukey *post-hoc* adjustments performed when significant differences were detected. Categorical variables were compared using the *X*^2^ test, as indicated. Correlation between variables was analyzed by linear regression analysis for continuous variables and by logistic regression analysis for categorical variables. A *p*-value < 0.05 was considered statistically significant. The statistical analysis was done using SPSS software, version 21.0 (IBM Corporation, NY, USA).

## Results

### Clinical Characteristics

Patients (*n* = 59; mean age, 65.5 ± 9.2 years) who met the baseline inclusion criteria and from whom adequate image quality with optimal rational tracking of any myocardial segments was obtained were evaluated in this study. Patients were divided into two groups, those with insignificant CAD (*n* = 33; <70% diameter stenosis) according to angiography and those with significant CAD (*n* = 26; ≥70% diameter stenosis; mean age, 62.7 ± 10.6 years). Cardiac catheterizations were performed within 2–3 weeks following echocardiography. There were no significant differences in baseline parameters and clinical data between the insignificant and significant CAD groups, as presented in Table 1.

**Table 1:** Comparison of patient characteristics data in the insignificant and significant CAD groups.

	**Insignificant CAD**	**Significant CAD**	***p-*** **value**
	**(*n* = 33)**	**(*n* = 26)**	
Gender (men/women), *n*	16/17	16/10	0.982
Age (years)	66.4 ± 10.7	62.7 ± 10.6	0.186
Weight (kg)	65 ± 13.1	73.1 ± 15.3	0.091
Height (m)	1.6 ± 0.1	1.7 ± 0.1	0.498
BMI (kg/m^2^)	24.4 ± 3.6	26.5 ± 5	0.162
Smoking, *n* (%)	7(21)	4 (15)	0.142
Family history, *n* (%)	19 (58)	14 (54)	0.763
Diabetes, *n* (%)	12 (36)	13 (50)	0.361
Hypercholesterolemia, *n* (%)	8 (24)	2 (8)	0.063
Hypertension, *n* (%)	20 (61)	16 (62)	0.658
Total cholesterol (mg/dL)	169.8 ± 40.6	158.2 ± 37.4	0.878
LDL cholesterol (mg/dL)	106.3 ± 36.7	99.7 ± 33.5	0.575
HDL cholesterol (mg/dL)	52.2 ± 14.6	42.6 ± 11.1	0.720
Triglycerides (mg/dL)	120.8 ± 71.7	150.1 ± 71.6	0.422
BUN (mg/dL)	19.6 ± 8.2	21.6 ± 10.6	0.372
Creatinine (mg/dL)	0.9 ± 0.5	1.1 ± 0.5	0.657
eGFR (mg/dL)	79.4 ± 31.5	58.8 ± 30.4	0.091
Glucose (mg/dL)	118 ± 40.8	143.3 ± 96.8	0.133
HbA1c (%)	7.4 ± 3	8.0 ± 2.5	0.630
**Medications**			
Anticoagulant, *n* (%)	19 (58)	26 (100)	0.375
Nitrate, *n* (%)	6 (18)	7 (27)	0.789
β-blocker, *n* (%)	10 (30)	9 (35)	0.973
ARB, *n* (%)	7 (21)	3 (12)	0.595
CCB, *n* (%)	13 (39)	15 (58)	0.234
Statins, *n* (%)	9 (27)	15 (58)	0.649
Diuretics, *n* (%)	9 (27)	4 (15)	0.308

*LDL, low-density lipoprotein; HDL, high-density lipoprotein; BUN, blood urine nitrogen; eGFR, estimated glomerular filtration rate; HbA1c, glycohemoglobin; ARB, aldosterone receptor blockers; and CCB, calcium-channel blocker*.

*Data are presented as mean ± SD or number (percentage)*.

### Baseline Echocardiographic Findings

Conventional echocardiographic data are shown in Table 2. No patient had evidence of RWMA, and all patients had globally preserved and normal LVEF at admission, both in the insignificant (65.3 ± 4.8) and significant CAD groups (67.2 ± 4.9). There were no significant differences in echocardiographic parameters between patients with insignificant and significant CAD except interventricular septal width (IVSd). Patients with significant CAD had thicker IVSd than patients with insignificant CAD (1.1±0.3 vs. 0.9±0.2, respectively; *p* = 0.024).

**Table 2:** Comparison of conventional echocardiographic measurements in the insignificant CAD and significant CAD groups.

	**Insignificant CAD**	**Significant CAD**	***p*** **-value**
	**(*n* = 33)**	**(*n* = 26)**	
IVSd (cm)	0.9 ± 0.2	1.1 ± 0.3	**0.024**
LVIDd (cm)	3.8 ± 0.4	4.0 ± 0.6	0.196
LVPWd (cm)	1.1 ± 0.3	1.2 ± 0.3	0.355
LVIDs (cm)	2.6 ± 0.3	2.7 ± 0.3	0.404
LVEF (%)	65.3 ± 4.8	67.2 ± 4.9	0.138
E vel (m/s)	0.7 ± 0.2	0.7 ± 0.2	0.914
A vel (m/s)	0.9 ± 0.2	0.8 ± 0.3	0.070
E/A ratio	0.8 ± 0.3	1.2 ± 0.9	0.061
e' (cm/s)	7.1 ± 2.4	6.8 ± 2.0	0.621
E/e'	11.0 ± 4.0	11.7 ± 4.4	0.572
GLS (%)	−16.7 ± 4.7	−15.1 ± 5.0	0.140
GLSR (1/s)	1.0 ± 0.4	0.9 ± 0.3	0.294

*IVSd, intraventricular septal width in diastole; LVIDd, left ventricular Internal dimension in diastole; LVPWd, left ventricular posterior wall width in diastole; LVIDs, left ventricular Internal dimension in systole; LVEF, left ventricular ejection fraction; E vel, early mitral flow velocity; A vel, late mitral flow velocity; E/A, ratio of mitral peak velocity of early filling (E) to peak velocity of late filling (A); E/e', ratio of mitral peak velocity of early filling (E) to early diastolic mitral annular velocity (e'); GLS, global longitudinal strain; and GLSR, global longitudinal SR*.

*Data are presented as mean ± SD or number (percentage)*.

*The bold value indicate statistically significant analysis, p-value < 0.05*.

### 2D-STE Analysis

**Figure 3:**
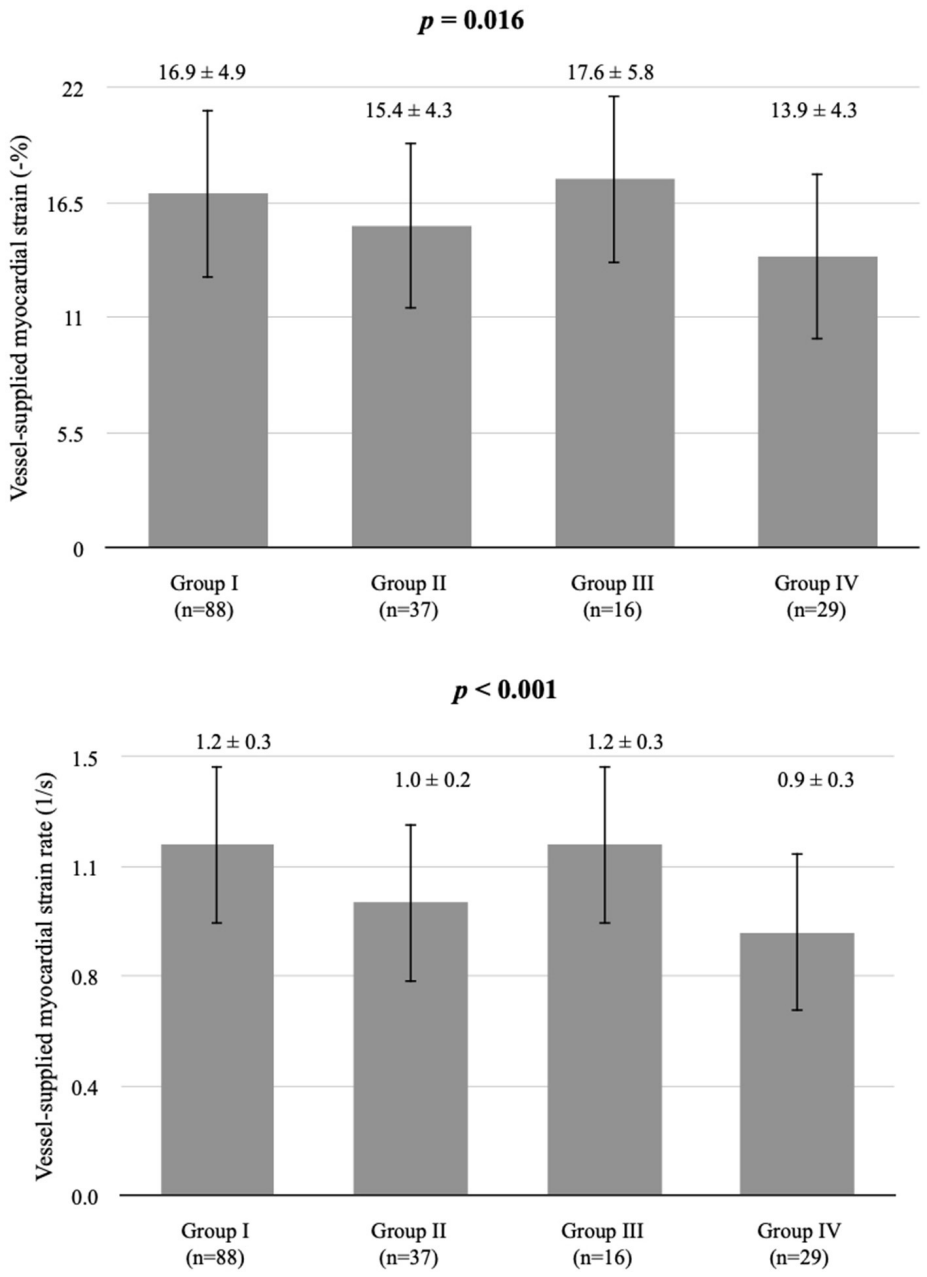
Comparison of longitudinal strain and SR in vessel-supplied myocardium according to coronary artery stenosis severity. ^*^Group I, mild, stenosis ≤ 25%; group II, moderate, stenosis 26–49%; group III, severe, stenosis 50–74%, and group IV, very severe, stenosis ≥75% following criteria modified from Arbab-Zadeh and Fuster (22, 23). Vessel-supplied myocardial strain is presented as a percentage. Vessel-supplied myocardial SR is in the unit of 1/s.

We examined 177 vessels in 59 patients. Among these vessels, seven had hypoplasia. When a vessel had hypoplasia, the myocardium segments supplied by the nearby coronary artery were combined with the adjacent myocardial strain and SR of the adjacent coronary arteries. Therefore, the VMS and VMSR areas were adjusted accordingly. The representative cases of longitudinal strain and SR (Figure 2) with quantification using EchoPac software are presented. The global longitudinal strain (GLS) and SR (GLSR) were initially quantified. However, the GLS and GLSR in the insignificant CAD group were not significantly different from those in the significant CAD group, *p* = 0.140 and *p* = 0.294, respectively. The longitudinal strain and SR of the vessel supplied areas in each stenosis severity group showed significant differences between groups, as determined by one-way ANOVA [*F*_(3,169)_ = 3.53, *p* = 0.016] and [*F*_(3,169)_ = 6.86, *p* < 0.001], respectively (Figure 3). The VMSs and VMSRs in each group were compared. A Tukey's HSD *post-hoc* test revealed that the longitudinal strain in vessels with very severe stenosis (group IV; *n* = 29), 13.9 ± 4.3, was significantly smaller than that in vessels with mild stenosis (group I; *n* = 88), 16.9 ± 4.9, *p* = 0.023. For the longitudinal SR, vessels with very severe stenosis (group IV; *n* = 29), 0.9 ± 0.3, and moderate stenosis (group II; *n* = 37), 1.0 ± 0.2, showed significantly smaller values than that of vessels with mild stenosis (group I; *n* = 88), 1.2 ± 0.3, *p* < 0.001 and *p* = 0.021, respectively (Table 3). The results implied that vessels with more severe stenosis have lower SR values. Furthermore, the results demonstrated that VMSR is superior to VMS as a sensitive predictor of coronary artery condition. The inter-observer variabilities for the measurements of GLS and GLSR were 95.8 and 96.4%, respectively, and those for segmental strain and SR were 97.3 and 95.2%, respectively.

**Table 3:** Longitudinal strain and SR according to coronary artery stenosis severity.

	**Stenosis (I)**	**Stenosis (J)**	**Mean difference (I-J)**	***p*** **-value**
	I	II III IV	1.4 ± 0.9 0.7 ± 1.3 2.9 ± 1.0	0.415 0.944 **0.023**
Longitudinal strain				
	II	III IV	2.1 ± 1.4 1.5 ± 1.1	0.430 0.587
	III	IV	3.6 ± 1.4	0.069
	I	II III IV	0.18 ± 0.06 0.02 ± 0.09 0.28 ± 0.07	**0.021** 0.997 ** <0.001**
Longitudinal SR	II	III IV	0.17 ± 0.09 0.09 ± 0.08	0.320 0.645
	III	IV	0.26 ± 0.10	0.052

*The bold values indicate statistically significant analysis, p-value < 0.05*.

## Discussion

This cross-sectional study prospectively recruited patients diagnosed with CAD. According to previous studies, longitudinal myocardial strain is the most clinically relevant and reproducible index among all cardiac dimensional deformation (24, 25). We focused on the correlation between regional myocardial deformation and coronary artery stenosis to determine the potential of 2D-STE as an early diagnostic tool for CAD. Here, the VMS and VMSR were evaluated. The identification of CAD is accomplished by the aforementioned clinical protocols. No one tool is perfect for diagnosis. Thus, a combination of various methods is required to identify CAD precisely. The ideal utility is approachable, non-invasive, cost-effective, none/less radiative with high sensitivity and specificity. 2D-STE fulfills all these requirements. To the extent of accurate CAD diagnosis, presentation of the clinical symptoms, checking the laboratory data, baseline electrocardiogram test (ECG), exercise stress tests (treadmill exam) and echocardiography are routinely examined. On condition that using the combination of baseline evaluations could not verify CAD, computed tomography angiography and nuclear medicine procedures are required. Stefanini et al. described that the sensitivity and specificity of CT angiography to detect coronary diameter stenosis >50% are ~81–99%, and 64–93%, respectively. However, CT angiography has more clinical role in patients with low to intermediate CAD risk, compared to catheterization exam. The specificity and negative predictive value decrease once patient risk increases (26). For nuclear thallium stress tests, the accuracy for detecting CAD was reported that the sensitivity varies from 70 to 92%, whereas the specificity is around 70% (27). Various analysts described that sensitivity of the diagnostic power of treadmill stress testing ranges from 61 to 73%, while specificity is around 59–81% (28, 29). For global longitudinal strain, the sensitivity of CAD diagnostic rate is 74.4%, and specificity is 72.1% (30). Given all the above data, the role of STE in CAD diagnosis is superior to treadmill stress testing, equivalent to nuclear thallium stress tests, and inferior to CT angiography. Nonetheless, CT angiography has limited roles in general practice. Echocardiography could be easily accessible and routinely performed in patients with suspected CAD. From all of the above factors, STE becomes a promising tool for diagnosis of CAD. Although these alternative methods have relatively higher sensitivity and specificity, the arrangements are time-consuming, costly, require radiologists, technicians of nuclear medicine involvement, more health insurance reimbursement and delayed diagnosis. Besides, computed tomography angiography and nuclear medicine procedures have been concerned to be relative risk due to radiation exposure from cumulative imaging examinations. The application of 2D-STE demonstrated that early identification of CAD by echocardiography was a primarily credible diagnostic procedure non-invasively and less costly. The establishment of 2D-STE status contributes the “assisted technologies in diagnostic decision making” to be possible in CAD identification. STE data could be immediately proceeded following echocardiography by software, which reveals the possibility of vessel stenosis. Accordingly, the treatment plan is made. STE technique could early differentiate vessels with non or mild stenosis from the vessels with high possibility of severe stenosis which require further evaluation by angiography or angioplasty. Our modality of diagnostic technology proposed that the STE technique could be used to screen the condition of vessel stenosis in CAD time-efficiently and primarily in an economic way without performing unnecessary CT or nuclear medicine. Even though 2D-STE implementation requires investment in the technology including ultrasound and STE software, CT and nuclear medicine machines are much more costly. Since quality image acquisition is significant for analysis, capacity building of the human resource is important. The technique trainings and experience exchanges are needed to provide to physicians and sonographers for STE implementation and measurement. Our study elucidated the segmental tissue speckle tracking could predict stenosis severity of specific coronary arteries in patients with CAD, which provide clinical impression for early intervention and efficient treatment to prevent acute illness condition and more complex treatment thereafter.

GLS can be used early to identify patients with CAD based on findings of a previous study (31). However, we found that in patients without RWMA, GLS and GLSR were not able to differentiate significant from insignificant CAD. Classification of patient groups by significant (>70% stenosis) and insignificant (<70% stenosis) CAD in this study aimed to elucidate and decrease the confounding factors such as hypertension and diabetes mellitus, which impact on strain and SR. These factors were similar (*p* > 0.05) in both groups. Thus, these confounding factors will not affect our analysis of VMS and VMSR. This is the first study that reveals that VMS and VMSR serve as better indicators for the evaluation of CAD than global myocardium tissue speckle tracking. The deformation of the myocardium supplied by the three main arteries, as mentioned earlier, was comprehensively quantified. Our analysis demonstrated that segmental longitudinal strain and SR values of the particular vessel-supplied myocardium significantly decreased corresponding to the severity of vessel stenosis. In general, the longitudinal strain and SR of the vessel supplied areas in vessels with mild and very severe stenosis have demonstrated significant differences. This implies that VMS and VMSR are feasible as early diagnostic parameters of CAD at high risk of myocardial ischemia. The lower VMS and VMSR, the higher possibility of severe coronary stenosis is. The VMS and VMSR lower than 13.9 ± 4.3 and 0.9 ± 0.3, respectively predicted the severe coronary stenosis. The VMS and VMSR higher than 16.9 ± 4.9 and 1.2 ± 0.3, respectively predicted mild or no coronary artery stenosis. In our finding, VMSR correlated even more with coronary artery stenosis severity. This elucidates that VMSR is a sensitive predictor of coronary artery condition. It will have a promising role in the evolution of diagnostic parameters. Our results indicated that VMSR was generally superior to VMS. Previous studies in patients with CAD reported that the impairment of myocardium deformation, specifically strain evaluated by 2D-STE, initially occurs from the endocardial layer. The endocardial layer of the myocardium is more vulnerable to ischemia (32). Compared to strain, which is load-dependent, SR is less related to pre-load and after-load (33). Moreover, SR imaging with elastance has shown a strong correlation with peak and mean SRs, higher than conventional tissue velocity or strain (34). However, one pitfall of SR measurement was noise sensitivity. The SR calculation influences the signal-to-noise ratio (35). Tissue tracking techniques have been evolved for two decades. There are technical challenges in STE that will have to be overcome (36). For instance, LV global transitional motion is the cause of baseline drift. The measurement of displacement may not be zero at the end of the cardiac cycle (i.e., from breathing, which is not synchronized with the cardiac cycle). To correct the unwanted calculation error, vendors have suggested that users apply drift compensation (37, 38). Despite that, our study showed good inter-observer reproducibility, as mentioned above. This is due to the improvement of the machinery and software for speckle tracking algorithms that influence the reduction of this variation. As another example, calculations for all three myocardial layers contribute to the strain and SR being more reliable and reproducible. Imaging system validation could facilitate good quality images. In terms of resolution, the number of beams per image sector, pixel size (spatial) and acquisition frame rate (temporal) setting considerably improve image quality (39). We optimized the frame rate for the best tracking and circumspectly decreased the spatial resolution to elevate the accuracy of the tracking technique. To evaluate the VMS and VMSR precisely, our study minimized the risk of comorbidity effects by excluding confounding factors of heart wall motions, diabetes, and hypertension, which may influence cardiac function. In addition, patients with conditions of systolic dysfunction and RWMA were also excluded, because VMS and VMSR can be affected as well, especially in chronic patients.

## Limitations

This was a small-scale, single-center study and the first of its kind. A multicenter study with a larger number of patients should be pursued to investigate this issue further. In addition, we excluded patients with RWMA. Therefore, our method can be applied only to patients without RWMA. However, we demonstrated the efficacy of VMS and VMSR. Finally, different machine vendors could be assessed, and their results compared among. Standardization among different vendors and software applications is required to improve the technical pitfalls for more efficient clinical meaning and application for all 2D-STE.

## Conclusion

Our findings revealed that strain and SR of the myocardium supplied by LAD, LCX, and RCA could be used to predict the stenosis condition in each coronary artery. The actual stenosis rate in catheterization proved that this technique is a potentially valuable clinical tool to assess coronary artery condition and implied the application of this non-invasive method of tissue speckle tracking for early evaluation and diagnosis of CAD.

## Data Availability Statement

The original contributions presented in the study are included in the article/supplementary material, further inquiries can be directed to the corresponding author/s.

## Ethics Statement

The studies involving human participants were reviewed and approved by the Institutional Review Board of Taipei City Hospital (TCHIRB-1020802-E). The patients/participants provided their written informed consent to participate in this study.

## Author Contributions

SC contributed to investigation, data curation, formal analysis, and writing original draft. S-JC contributed to conceptualization, methodology, resources, review, editing, and supervision. MD provided resources, validation, supervision, data curation, review, editing, and funding acquisition. S-CC contributed to validation and investigation. C-LC and C-YH contributed to investigation and resources. H-HC and C-LT provided supervision. All authors contributed to the article and approved the submitted version.

## Funding

This work was supported in part by a Grant-in-Aid for Scientific Research C (21K12701) from the Japan Society for the Promotion of Science (MD). This work was also supported by the faculty of the Graduate Institute of Biomedical Materials and Tissue Engineering, Taipei Medical University, Division of Cardiology, Department of Internal Medicine, Taipei City Hospital Yangming Branch, Taiwan, and Department of Cardiovascular Medicine, The University of Tokyo Hospital, Tokyo, Japan.

## Conflict of Interest

The authors declare that the research was conducted in the absence of any commercial or financial relationships that could be construed as a potential conflict of interest.

## Publisher's Note

All claims expressed in this article are solely those of the authors and do not necessarily represent those of their affiliated organizations, or those of the publisher, the editors and the reviewers. Any product that may be evaluated in this article, or claim that may be made by its manufacturer, is not guaranteed or endorsed by the publisher.
